# Ambiguity in Statistical Analysis Methods and Nonconformity With Prespecified Commitment to Data Sharing in a Cluster Randomized Controlled Trial

**DOI:** 10.2196/54090

**Published:** 2024-04-03

**Authors:** Yasaman Jamshidi-Naeini, Lilian Golzarri-Arroyo, Deependra K Thapa, Andrew W Brown, Daniel E Kpormegbey, David B Allison

**Affiliations:** 1 Department of Epidemiology and Biostatistics School of Public Health Indiana University Bloomington Bloomington, IN United States; 2 Department of Biostatistics University of Arkansas for Medical Sciences Little Rock, AR United States; 3 Arkansas Children's Research Institute Little Rock, AR United States

**Keywords:** cluster randomized trial, clustering, nesting, data availability, random allocation, data sharing, reproducibility

In a published cluster randomized controlled trial (cRCT) [1] on the effects of desks on physical behaviors, 66 individuals were randomized into 3 groups by their office space (ie, clusters): the seated desk control (n=21; 8 clusters), sit-to-stand desk (n=23; 9 clusters), or treadmill desk (n=22; 7 clusters) group.

In the article, there is ambiguity regarding whether clustering (potential nonindependence of observations within the same office space) and nesting (due to the hierarchical structure of the data; see definitions from Jamshidi-Naeini et al [2]) have been accounted for. Furthermore, it appears that the data underlying the published results are not available to other researchers, which is contrary to the journal’s policy indicating “a submission to JMIR journals implies that…all relevant raw data, will be freely available to any researcher wishing to use them for non-commercial purposes….”

The description of methods indicates using random intercept mixed linear models accounting for repeated measures and clusters. However, it is stated elsewhere that “[t]he cluster effect did not significantly (all *P* values >.05) account for the variability in any of the outcome variables….Therefore, aim 1 and aim 2 outcome observations…were analyzed at the participant level instead of cluster…” [1].

The statement regarding accounting for the clustering effect is ambiguous. If the authors ignored the clustering effect based on the reasoning that participant outcomes within the same cluster are unrelated, as indicated by intraclass correlation coefficient (ICC) values that one chooses to describe as “small” or nonstatistical significance of the ICC values at some nominal α level (eg, 0.05), such reasoning is erroneous. A sample ICC or its associated *P* value is not an appropriate metric on which to determine whether one should account for clustering. Ignoring clustering, regardless of a sample ICC’s magnitude or associated *P* value, potentially leads to miscalculation of variance components and type I error rates above the nominal significance level [3,4].

In a cRCT with such unequal cluster sizes (ranging from 1 to 11 participants), there is no exact size *α* test, and type I error inflation may occur. Therefore, to ensure control of type I error rate, it is essential to apply appropriate weighting for unequal cluster sizes. In addition, the nesting effect that arises from the hierarchical structure of the data in cRCTs was not considered in the statistical analyses. Adjusting *df* (eg, by between-within determination [4]) could have accounted for this nesting effect.

We requested the raw data to reproduce the analyses (see definition of “reproducing” in *Reproducibility and Replicability in Science* [5]) and potentially use alternative corrected methods to reanalyze the data. Data were not shared with us. The authors stated that this decision was made “to ensure the integrity of ongoing research being conducted using the same dataset.” Nevertheless, sharing data for the purpose of reproducing published results does not compromise the integrity of further analyses on the same data set. Withholding data, on the other hand, renders the study irreproducible and thus compromises the trustworthiness of the published results.

The concerns raised herein should be addressed to ensure the integrity, transparency, and reproducibility of the published findings.

## Abbreviations

cRCT: cluster randomized controlled trial
ICC: intraclass correlation coefficient
